# Statistical framework to support the epidemiological interpretation of SARS-CoV-2 concentration in municipal wastewater

**DOI:** 10.1038/s41598-022-17543-y

**Published:** 2022-08-05

**Authors:** Xiaotian Dai, David Champredon, Aamir Fazil, Chand S. Mangat, Shelley W. Peterson, Edgard M. Mejia, Xuewen Lu, Thierry Chekouo

**Affiliations:** 1 Department of Mathematics and Statistics, University of Calgary, Calgary, AB Canada; 2 Public Health Risk Sciences Division, National Microbiology Laboratory, Public Health Agency of Canada, Guelph, ON Canada; 3 One Health Division, National Microbiology Laboratory, Public Health Agency of Canada, Winnipeg, MB Canada

**Keywords:** Computational models, Statistical methods, Viral infection

## Abstract

The ribonucleic acid (RNA) of the severe acute respiratory syndrome coronavirus 2 (SARS-Cov-2) is detectable in municipal wastewater as infected individuals can shed the virus in their feces. Viral concentration in wastewater can inform the severity of the COVID-19 pandemic but observations can be noisy and sparse and hence hamper the epidemiological interpretation. Motivated by a Canadian nationwide wastewater surveillance data set, unlike previous studies, we propose a novel Bayesian statistical framework based on the theories of functional data analysis to tackle the challenges embedded in the longitudinal wastewater monitoring data. By employing this framework to analyze the large-scale data set from the nationwide wastewater surveillance program covering 15 sampling sites across Canada, we successfully detect the true trends of viral concentration out of noisy and sparsely observed viral concentrations, and accurately forecast the future trajectory of viral concentrations in wastewater. Along with the excellent performance assessment using simulated data, this study shows that the proposed novel framework is a useful statistical tool and has a significant potential in supporting the epidemiological interpretation of noisy viral concentration measurements from wastewater samples in a real-life setting.

## Introduction

The ribonucleic acid (RNA) of the severe acute respiratory syndrome coronavirus 2 (SARS-CoV-2) is detectable in municipal wastewater as infected individuals can shed the virus in their feces^1,2^. The National Microbiology Laboratory (NML) at Public Health Agency of Canada (PHAC), in partnership with Statistics Canada, is actively monitoring SARS-CoV-2 virus concentrations in wastewater treatment plants (WWTP) across major Canadian cities in order to inform public health actions.

Wastewater surveillance has proven to be a useful tool for disease outbreak monitoring before the COVID-19 pandemic^3,4^. A number of research groups have studied the COVID-19 pandemic from the perspective of wastewater-based epidemiology: Ling et al.^1^ detected viral RNA in patients’ urine and fecal samples; Medema et al.^2^ reported that SARS-CoV-2 RNA was present in sewage at the beginning of the COVID-19 pandemic in Netherlands; Ahmed et al.^5^ also reported the detection of SARS-CoV-2 in wastewater plants in Australia; Peccia et al.^6^ monitored the viral RNA concentrations in primary sewage sludge in the New Haven (Connecticut, USA) and claimed the viral RNA concentrations to be a leading indicator of the rise and fall in the number of positive clinical cases and local COVID-19 hospital admissions; Acosta et al.^7^ assessed the numerical relationship between hospitalized COVID-19 cases and SARS-CoV-2 RNA gene-targets (N1 and N2) in the wastewater from three adult tertiary-care hospitals in Calgary (Alberta, Canada).

Compared to previous COVID-19 wastewater-based epidemiology research, our study depends on a large-scale and nationwide collection of municipal wastewater at 15 sampling sites across five Canadian cities: Edmonton, Halifax, Montréal, Toronto, and Vancouver. This PHAC program relies on wastewater surveillance to monitor trends in SARS-CoV-2 prevalence and track community infections throughout the COVID-19 pandemic. The wastewater surveillance data were collected from 15 WWTPs in the five cities starting from October, 2020 up until November, 2021. We focus on developing a novel and accurate statistical framework to model the dynamic trajectory of SARS-CoV-2 concentration in wastewater. There are two main challenges in the interpretations of wastewater-based epidemiology signals. The first challenge is that the viral concentrations in wastewater samples can be influenced by many known and unknown factors. The known factors are sample storage temperature^8^, WWTP influent volume^7^, in-sewer processes such as the presence of sewer biofilms^9,10^ to name a few. The unknown factor includes but not limited to the unavoidable technical and statistical errors in wastewater sampling and experimental replicates. The main research question here is that whether increases in wastewater viral concentration measurements indicate a significant increase of viral shedding and potentially a disease outbreak in the region serviced by a particular WWTP. In this article, the proposed framework accounts for the effects of known factors by incorporating them as covariates in a joint statistical model. Moreover, in order to reduce the influence of unknown factors, the measurements of viral concentrations is done on multiple technical replicate samples. The proposed framework also uses a method called the functional principal component analysis^11,12^, a state-of-the-art statistical method developed for analysing curve data, to detect the major trends in viral concentration trajectory while removing small fluctuations that are likely to be caused by technical errors. The second challenge of interpreting wastewater-based epidemiology signals is that the wastewater samples are usually taken and measured on sparse and irregular time intervals. For example, the PHAC program samples wastewater data from 15 WWTP sites across Canada, and each PHAC-associated lab have different weekly schedule of taking samples and reporting viral concentrations. The data generated from longitudinal wastewater monitoring studies cannot be directly interpreted without appropriate statistical interpolations^6^. In this article, the proposed framework applies the functional principal component analysis for sparse longitudinal observations^12^ to interpolate dense and regularly-observed viral concentrations through detecting trends and borrowing information across WWTP sites.

For each wastewater sampling site, our framework aims to answer two specific questions: (1) How to detect true signals of viral concentration increases/decreases out of noisy observations? In order to better inform public health actions, a more reliable interpretation of wastewater-based epidemiology trend is needed. The accurate detection of trend change depends on the proposed framework to tackle the two challenges described above. Also, we use the Markov chain Monte Carlo (MCMC) framework^13^ to estimate the probability of an increase or decrease in true viral concentrations. The second question is (2) How to accurately forecast the future trajectory of viral concentrations in wastewater? The proposed framework is employed to detect the true trends of viral concentration out of noisy and sparse observations and to forecast the future trajectory of viral concentrations in wastewater. These capabilities are demonstrated through simulated data and the Canadian nationwide wastewater surveillance program data. Due to the scale of the data collection and the support from the PHAC, the proposed framework is already having real-life impacts on pandemic monitoring and can be widely applied in future epidemiology studies.

## Materials and methods

### Wastewater sampling and SARS-CoV-2 concentrations

Wastewater samples were collected approximately twice a week at each sampling location. Sample collection dates may differ by location. Influent samples were collected from WWTPs in each city. For Vancouver, the plants sampled are located in Annacis Island (VAI), Iona Island (VII), Lions Gate (VLG), Lulu Island (VLI); for Edmonton at Gold Bar (EGB); for Toronto at Ashbridges (TAB), Highland Creek (THC), Humber (THU) and North Toronto (TNT); for Halifax at Dartmouth (HDA), Halifax Downtown (HHA), Millcove (HMC). For Montréal, the sampling locations were not at the municipal WWTP but at two locations on the Island of Montréal, here labelled Montréal North (MMN) and Montréal South (MMS), each covering approximately one half of the population of the island. Wastewater samples were collected at the sampling site and then shipped to NML in Winnipeg (Manitoba, Canada) for analysis.

Viral RNA present in the wastewater samples was quantified using the reverse transcription-quantitative polymerase chain reaction (Rt-qPCR) test with the United States Centers for Disease Control and Prevention (US-CDC) N1 and N2 primers using the method described in Nourbakhsh et al.^14^. For all the wastewater samples, the N1 and N2 gene concentrations are measured by two technical replicates.

### Statistical model

For the purpose of data quality assurance, each wastewater sample is measured with two technical replicates. The virus concentration values are observed on an irregular time grid as samples were not collected on the same days between locations. The curves of concentration values for 15 sampling sites need to be imputed and mapped onto a consistent and regular time grid, so that a continuous trend of viral concentration can be estimated and different curves are comparable to each other. Also, the curves of virus concentration values can be affected by errors associated with technical replicates. Functional principal components analysis (FPCA)^12^ and the extension of FPCA to include covariates^15^ can solve the issues mentioned above by: (1) leveraging the correlations among a group of curves; (2) imputing missing values of the curves on a regular time grid; (3) estimating a smoothed mean curve and eigenfunctions from a group of noisy curves, with the eigenfunctions representing and explaining direction of variability (see Yao et al.^12^ for details); and (4) incorporating and estimating the effects of covariates (e.g., sample storage temperature, daily influent volume at the wastewater treatment plant) on viral concentrations as fixed effects in a joint regression-like model^15^.

For each sampling site and target gene (N1 and N2), the full model is written as

1 $$\begin{aligned} Y_{ik}(T_{it}) = Y_{itk}= \mu (T_{it}) + \sum _{p=1}^{P} \beta _p(T_{it}) X_{ip}(T_{it}) + \sum _{l_0=1}^{L_0} \xi _{i{l_0}} \phi _{l_0}(T_{it}) + \varepsilon _{itk}, \end{aligned}$$

where *i* is the index of a sampling site (i.e., $i=1,\ldots ,15$), *t* is the index of a sample taken at site *i* (i.e., $t=1,\ldots ,N_i$, $N_i$ is the number of time points (daily) for site *i*), and *k* is the index of a technical replicate, i.e., $k=1,2$. $\mu (T_{it})$ is the overall mean function of all sites and technical replicates at time $T_{it}$, which is the time of when the *t*th sample at site *i* is taken. $\varepsilon _{itk}$ is the error contained in each technical replicate *k*, site *i* and sample *t*. All the errors are assumed to follow an independent and identical normal distribution with $E(\varepsilon _{itk}) = 0$ and $\text {var}(\varepsilon _{itk}) = \sigma ^2$. $\beta _p(T_{it})$ is the time-varying effect of the *p*th covariate at time $T_{it}$, and $X_{ip}(T_{it})$ is the observed value of the *p*th covariate at time $T_{it}$. When $P=0$, Eq. (1) is reduced to a model without covariates. $L_0$ is the number of basis functions extracted from the FPCA process. A basis function of a principal component (PC) can explain certain direction of variation in the observed curves, with the first $L_0$ PCs covering a desirable proportion of the total variation (e.g., 90% of total variation). $\phi _{l_0}$ is the estimated basis function associated with the $l_0$th PC. Parameters $\xi _{i{l_0}}$’s are random FPCA scores with $E(\xi _{i{l_0}}) = 0$ and $\text {var}(\xi _{i{l_0}}) = \lambda _{l_0}$, where $\lambda _{l_0}$ is the eigenvalue of the $l_0$th PC. In the FPCA process defined by^12^, an observed curve can be approximated by a linear combination of basis functions, with FPCA scores as the coefficients and eigenvalues as the variance of the FPCA scores. In this study, $\lambda _{l_0}$ is a random variable estimated along with $\xi _{i{l_0}}$’s.

To model the time-varying effect of the *p*th covariate $\beta _p(T_{it})$, we map the theoretically infinite-dimensional time-varying effect onto a system of basis functions and use the coefficients of these functions as the inputs of a joint regression. For convenience, we use the eigenfunctions derived from $X_{ip}(T_{it})$ curves as the basis functions here:

$$\begin{aligned} \beta _p(T_{it})= & {} \sum _{l_p=1}^{L_p} b_{l_p} \phi _{l_p}(T_{it}), \\ X_{ip}^*(T_{it})= & {} \sum _{l_p=1}^{L_p} x_{il_p} \phi _{l_p}(T_{it}), \end{aligned}$$

where $X_{ip}^*(T_{it})$ is a smoothed approximation of $X_{ip}(T_{it})$ derived from the FPCA process. By replacing $X_{ip}(T_{it})$ with $X_{ip}^*(T_{it})$ in Eq. (1), the time-varying effect of the *p*th covariate can then be represented by a vector of $L_P$ values: ${\varvec{b}}_p = \{ b_{1}, \ldots , b_{l_p}, \ldots , b_{L_p} \}$. Therefore, the full model in Eq. (1) can be rewritten as

2 $$\begin{aligned} Y_{ik}(T_{it}) = Y_{itk}= \mu (T_{it}) + \sum _{p=1}^{P} \sum _{l_p=1}^{L_p} b_{l_p} \{ X_{ip}^*(T_{it}) \phi _{l_p}(T_{it}) \} + \sum _{l_0=1}^{L_0} \xi _{i{l_0}} \phi _{l_0}(T_{it}) + \varepsilon _{itk}. \end{aligned}$$

We adopt a Bayesian framework for model estimation and inference via Markov Chain Monte Carlo (MCMC) sampling^16^. Prior distributions of unknown parameters are defined as follow:

- The variance of errors $\sigma ^2$ is assumed to follow an inverse Gamma distribution $\text {InverseGamma}(\alpha _{\sigma }, \beta _{\sigma })$, where $\alpha _{\sigma }$ and $\beta _{\sigma }$ are small values. This prior is non-informative that is we have little prior information about the parameter;
- We assume $\xi _{i{l_0}}$ follows a normal distribution with mean 0 and variance $\lambda _{l_0}$ that is $\xi _{i{l_0}} \sim \text {Normal}(0, \lambda _{l_0})$;
- The variance $\lambda _{l_0}$ follows an inverse Gamma distribution with shape and sclae parameters $\alpha _\lambda ^1$ and $\alpha _\lambda ^2$ respectively that is $\lambda _{l_0} \sim \text {InverseGamma}(\alpha _\lambda ^1, \alpha _\lambda ^2)$. We choose small values for $\alpha _\lambda ^1$ and $\alpha _\lambda ^2$, so the prior distribution is essentially non-informative. We note that $\phi _{l_0}$ is estimated from centered observed curves (i.e., $\mu (T_{it})$ was subtracted), and the term $\sum _{l_0=1}^{L_0} \xi _{i{l_0}} \phi _{l_0}(T_{it})$ is a zero-mean random process.
- Prior for ${\varvec{b}}_p$: To avoid overfitting, we regularize the coefficients vector ${\varvec{b}}_p$ by using the Bayesian group lasso penalty^17^. Specifically, the prior of ${\varvec{b}}_p$ follows a multivariate generalization of the double exponential distribution:
  $$\begin{aligned} {\varvec{b}}_p \propto \text {exp} \left( -\frac{\delta }{\sigma } || {\varvec{b}}_p ||_2 \right) , \end{aligned}$$
  where $|| {\varvec{b}}_p ||_2$ is the $L_2$ norm of ${\varvec{b}}_p$^18^, $\delta$ is a penalty parameter, and the double exponential distribution can be rewritten as a scale mixture of normal distribution with Gamma hyperpriors:
  $$\begin{aligned} {\varvec{b}}_p \sim \text {Normal}({\varvec{0}}, \tau _p^2 \sigma ^2 {\varvec{I}}_{L_p} ); \tau _p^2 \sim \text {Gamma}(\frac{L_p + 1}{2}, \frac{\delta ^2}{2}), \end{aligned}$$
  where ${\varvec{I}}_{L_p}$ is an identity matrix of dimension $L_p$.

Our MCMC algorithm will provide sample values of parameters and this will allow us to obtain sample values of ${\hat{Y}}_i(T_{it})$, estimate of the unobserved true concentration value. It’s formally defined as

3 $$\begin{aligned} {\hat{Y}}_i(T_{it}) = {\hat{\mu }}(T_{it}) + \sum _{p=1}^{P} \sum _{l_p=1}^{L_p} {\hat{b}}_{l_p} \{ X_p^*(T_{it}) \phi _{l_p}(T_{it}) \} + \sum _{l_0=1}^{L_0} {\hat{\xi }}_{i{l_0}} \phi _{l_0}(T_{it}). \end{aligned}$$

The replicate errors are essentially removed from the lab results. From the MCMC sample values of ${\hat{Y}}_i(T_{it})$, we can estimate the probability that today’s estimate of the true concentration value is larger than yesterday’s estimate. We denote these probabilities $Proba({\hat{Y}}_i(T_{it}) > {\hat{Y}}_i(T_{i,t-1}))$ which are estimated by the proportion of MCMC sample values that verify ${\hat{Y}}_i(T_{it}) > {\hat{Y}}_i(T_{i,t-1})$. In general, we can also estimate $Proba({\hat{Y}}_i(T_{it}) > {\hat{Y}}_i(T_{i,t-D}))$, where *D* is an arbitrary time difference of observations. For instance, when $D=7$, we look at the probability of weakly increase. We estimate these probabilities to conclude whether or not an increase (or decrease) in the observed virus concentration signals a significant increase (or decrease) in the true concentration.

To accurately forecast the future trajectory of viral concentration, we propose to use the autoregressive integrated moving average (ARIMA)^19^ to extend the smooth basis functions $\phi _{l_0}$’s (and $\phi _{l_p}$’s if there are covariates). The forecasts can be calculated based on the linear combination of basis functions shown in Eq.  (1).

We carry out our MCMC sampling by utilizing *Stan*^20^, a probabilistic programming language implemented in the R package *Rstan*^21^. The R package *refund*^22^ is used to compute eigenfunctions $\phi _{l_0}(T_{it})$’s. We create an R package called *WWmodel* for our framework. It is available on GitHub (https://github.com/xiaotiand/WWmodel).

## Results and discussion

### SARS-CoV-2 virus concentration modeling

**Figure 1:**
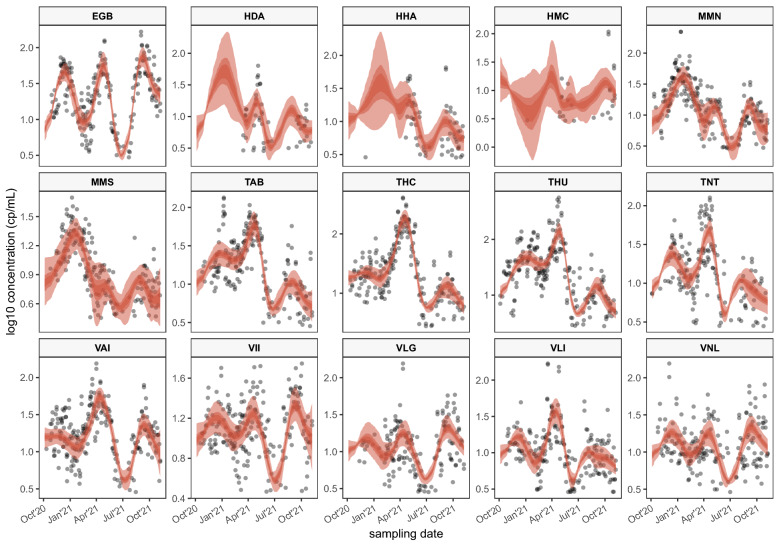
Black open circles represent the Log10 transformation of SARS-CoV-2 concentration observations for the N1 assay. Red shaded areas represent the range (lightest area), 50% (darkest area) and 80% credible intervals (slightly lighter area) of posterior curves at each date, from the model with covariates. Each panel represents a sampling location.

SARS-CoV-2 virus concentration are measured on N1 and N2 primer set (see “Wastewater sampling and SARS-CoV-2 concentrations”). For the N1 assay, the log base 10 transformation of virus concentrations of wastewater samples from 15 sampling locations are plotted in Fig. 1. As described in “Statistical model”, the proposed framework is based on a Bayesian model that can sample and estimate unknown parameters including ${\hat{Y}}_i(T_{it})$, the estimate of the unobserved true concentration value (see Eq. 3). ${\hat{Y}}_i(T_{it})$ can be seen as posterior estimates of the true concentrations after removing noises incurred by various factors. In this manuscript, for simplicity, we designate the posterior estimates of the true concentrations ${\hat{Y}}_i(T_{it})$ as the “posterior curves” generated from the proposed framework. For each site, the number of posterior curves is 2500, which corresponds to the number of MCMC iterations used for posterior inference. Figure 1 shows the distribution of posterior curves of ${\hat{Y}}_i(T_{it})$ at the 15 sampling locations using the comprehensive model with two covariates [(sample storage temperature in degree Celsius ($^{\circ }$C) and daily influent volume into the WWTP in megalitre (MI)], with color saturation representing the density of posterior curves. The two sampling sites at Halifax (HDA and HHA) do not report sampling results regularly, and the reported virus concentrations are very sparse for the two sites. However, thanks to the hierarchical structure of the proposed framework those sites borrow information from other sites; we can impute the “missing” concentrations, but the imputed posterior curves have a large variation and uncertainty, as shown in Fig. 1.

The root mean squared errors (RMSE) of the posterior curves at each site for the model without covariates (i.e. $P=0$) and the comprehensive model with two covariates are shown in Table 1. The RMSEs are calculated by comparing the posterior curves and the actual observations across 15 sampling sites. The standard errors of the RMSEs are also included in parenthesis. As shown in Table 1, the RMSE is slightly improved (lower) with the inclusion of two covariates, while the differences are not significant. This is probably due to the regularization of covariates’ effects, which makes the framework robust to the inclusion of a large number of candidate covariates (i.e., avoid overfitting). The modelling results of the primer gene N2 are included in the Supplementary Information (see Fig. S1).

**Figure 2:**
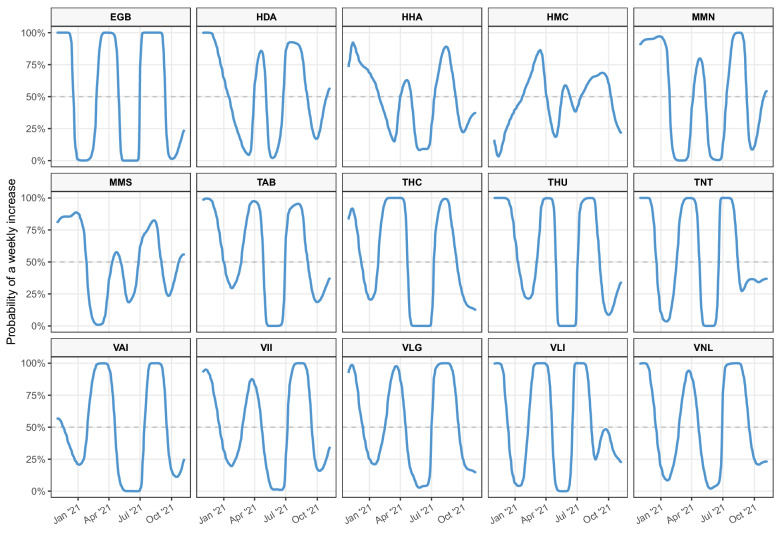
Time series of the probability of weekly increase, that is $Proba({\hat{Y}}_i(T_{it}) > {\hat{Y}}_i(T_{i,t-7}))$ ($D=7$) calculated with the comprehensive model (including covariates) for all sampling locations. The horizontal dashed line indicates the 50% probability.

As mentioned in “Statistical model”, the objective is to identify whether an increase in the observed virus concentration signals an increase in the true concentration. This is done by computing the probability to get an increase in true viral concentration from the past concentration at lag *D*: $Proba({\hat{Y}}_i(T_{it}) > {\hat{Y}}_i(T_{i,t-D}))$. Figure 2 shows the probability of an increase in the true concentrations for $D = 7$. The time lag *D* is in calendar days, so $D = 7$ means a timestamp difference of one calendar week. A high value of $Proba({\hat{Y}}_i(T_{it}) > {\hat{Y}}_i(T_{i,t-D}))$ (i.e., a value close to 1) suggests that there is a high chance of an increase in the true concentration value, and a low value of $Proba({\hat{Y}}_i(T_{it}) > {\hat{Y}}_i(T_{i,t-D}))$ (e.g., one close to 0) suggests that there is a high chance of a decrease in the true concentration value. On the other hand, a $Proba({\hat{Y}}_i(T_{it}) > {\hat{Y}}_i(T_{i,t-D}))$ value of around 0.5 suggests that the differences in the observed values at two timestamps are not significant. We also include the probability of an increase in the true concentrations for $D = 1$ and $D = 20$ (see Figs. S2, S3 in the Supplementary Information). As shown in Fig. 2, Figs. S2 and S3, the increasing signal will become clearer as the time lag *D* gets moderately larger (a large value of *D* may not be meaningful in practice). In Fig. 2, we can see that as we approach the end of the year 2021, we are also getting closer to the end of the third wave of the COVID-19 pandemic beginning October, 2020. At all locations, the probabilities strongly indicate an increase during the ascending phases of all three waves. In the future, the posterior estimates of true concentrations or the probabilities of increases can be used as a cleaner predictor of a spike of clinical cases rather than relying on observed concentrations which can be noisy as a result of technical and statistical errors^6,7^.

**Figure 3:**
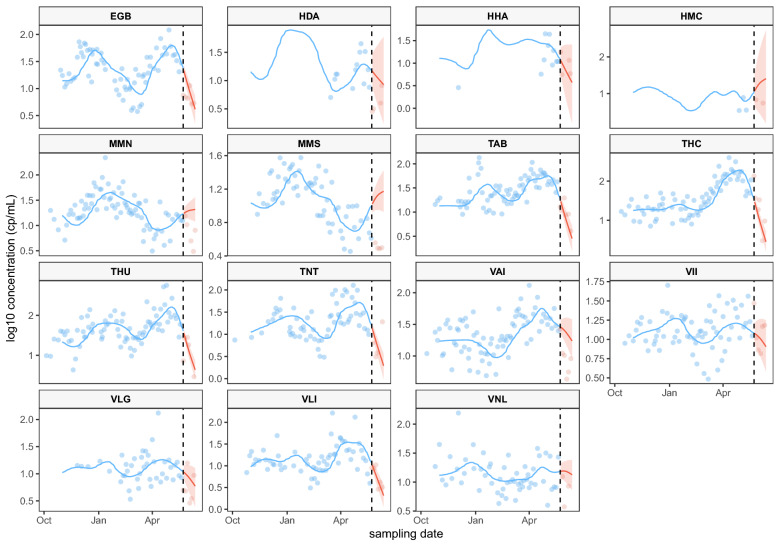
The historic data observed before May 23rd, 2021 (blue points) are used as training data and used to fit the full model (blue line represents the mean posterior curve). The mean posterior curve beyond the last observation date used for fitting is shown in red (red area for the 95% CrI). The red points represent the data forecasted. The forecasting horizon is one calendar month.

As detailed in “Statistical model”, the proposed framework can also be used to forecast future concentration values, and then produce signals for future pandemic waves simply by extending the basis functions $\phi _{l_0}(T_{it})$’s to future time points $T_{it} = T + 1, T + 2, \ldots$. To test the forecasting algorithm, virus concentrations of gene N1 during the calendar month after May 23rd, 2021 are held out and used as testing data. The historic N1 concentration values observed before May 23rd, 2021 are used as training data and used to build a forecasting model using our proposed framework. Figure 3 shows that, for the majority of the sampling locations, the forecasts are fairly predictive, 72% of the actual observations (blue dots) are within the range of the extended posterior curves (brown curves). Figure 3 suggests that the proposed framework can also successfully predict a downturn. The Supplementary Information shows another example of forecasting a new wave of the pandemic (see Fig. S6). In Fig. S6, the historic N1 concentration values observed before August 1st, 2021 are used as training data and used to build a forecasting model to forecast the concentrations in August, 2021.

**Table 1:** RMSE for both models. The standard errors of the RMSEs are in parenthesis. Larger standard errors indicate larger variations in posterior curves. This variation can be reflected in the widths of ribbons in Fig. 1.

	EGB	HDA	HHA	HMC	MMN
Model w/covariates	15.57 (0.01)	14.75 (0.07)	12.81 (0.04)	5.19 (0.03)	23.73 (0.01)
Model w/out covariates	14.75 (0.01)	12.79 (0.03)	10.47 (0.01)	4.08 (0.03)	22.05 (0.01)

	MMS	TAB	THC	THU	TNT
Model w/covariates	6.65 (0.00)	20.26 (0.01)	42.92 (0.05)	67.47 (0.08)	21.17 (0.02)
Model w/out covariates	6.56 (0.00)	20.16 (0.01)	38.91 (0.02)	63.01 (0.08)	21.13 (0.02)

	VAI	VII	VLG	VLI	VNL
Model w/covariates	19.18 (0.02)	9.34 (0.01)	17.48 (0.02)	22.99 (0.01)	16.52 (0.03)
Model w/out covariates	16.69 (0.01)	9.06 (0.00)	17.57 (0.02)	22.72 (0.01)	16.52 (0.03)

### Interpretation of wastewater-based epidemiology

According to Detsky and Bogoch^23^, the second and third waves of COVID-19 infections happened during the period September 2020 through August 2021. Here we investigate the pandemic waves from the perspective of the wastewater-based epidemiology. The number of weekly confirmed COVID-19 cases peaked in December, 2020 and April, 2021 in Canada^23^, which is consistent with the probability of weekly increase in wastewater viral concentrations for most major Canadian cities as shown in Fig. 2. The correlation between wastewater signals and reported cases may be weaker during the wave of the Omicron variant, as the clinical testing efforts have been scaled down across Canada and many other countries. This is why the interpretation of wastewater-based epidemiology has become more important for public health decision making and would be an indicator for disease outbreak.

The third wave of COVID-19 infections overwhelmed the capacity of healthcare system in Ontario province, Canada^23^, and the most populated city of Ontario is Toronto. This is reflected in Fig. 1 as the sampling sites TAB, THC, THU, and TNT all show a large spike of SARS-CoV-2 concentrations in wastewater samples around April, 2021. Improvement in infection control practices in long-term care facilities after the first two waves varied across Canada, with Quebec province showing significant improvement^23^, and the most populated city of Quebec is Montréal. This is also reflected in Fig. 1 as the sampling sites MMN and MMS show a smaller spike of SARS-CoV-2 concentrations in wastewater samples around April, 2021 compared to Toronto sites. The Atlantic provinces (including Halifax) fared very well due to its swift responses to new cases with rapid community tracing and testing^23^. As shown in Fig. 1, the wastewater signals observed by the three Halifax sites (HDA, HHA, and HMC) are very sparse, but the overall trends of the interpolated SARS-CoV-2 concentrations suggest that the viral concentrations in Halifax’s municipal wastewater are lower than those of other cities.

### Simulated data

Here we present a simulated data example using a statistical simulation design. Another simulated data example using an epidemic/mechanistic simulation design proposed by Nourbakhsh et al.^14^ is included in the Supplementary Information. See “Materials and methods” and Fig. S7 of the Supplementary Information.

The simulated data contain eight hypothetical sampling sites ($I = 8$). The basis functions ($\phi _{l_0}$) and eigenvalues are generated from the observed virus concentrations (on a log-scale) in the Canadian municipal WWTP samples. At each site and each time point, two replicates of measurements are simulated.

**Figure 4:**
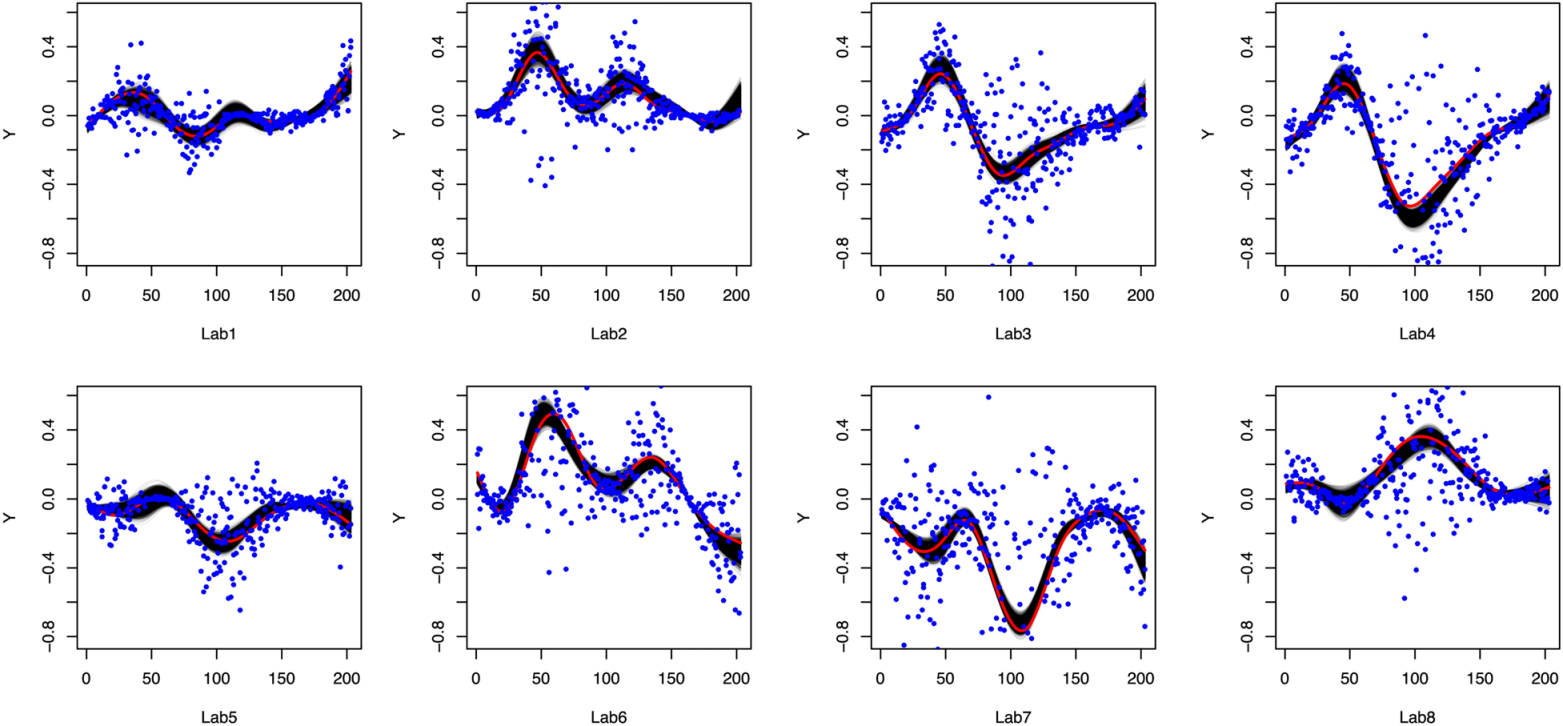
The simulation results of the statistical design with $\sigma _{it} / |\mu _{it}| = 1$. The posterior curves are in black, the truth line is a red line, and simulated observations are blue dots.

For each site, the observed concentration values are generated as:

$$\begin{aligned} Y_{ik}(T_{it}) = \mu _{it} + \varepsilon _{itk}, \end{aligned}$$

where $\mu _{it} = \sum _{l_0=1}^{4} \xi _{i{l_0}} \phi _{l_0}(T_{it})$, $\xi _{i{l_0}} \sim \text {Normal}(0, \lambda _{l})$, and $\varepsilon _{itk}$ is an independent error with $\varepsilon _{itk} \sim \text {Normal}(0, \sigma _{it})$. The standard deviation of the error term $\sigma _{it}$ is proportional to the true concentration $\mu _{it}$ (i.e., $\sigma _{it} / |\mu _{it}|$ is a constant). In other words, a larger concentration value can contain a larger observational error, which mimics the real-life situation. We vary $\sigma _{it} / |\mu _{it}| = 0.1, 0.5$ and 1 to test the robustness of the proposed framework with respect to the estimation of coefficients. Also, each simulated observed concentration $Y_{ik}(T_{it})$ has around 10% of missing data time points. Then we applied our framework to the simulated data for each sampling site. The simulated data and posterior curves (i.e., posterior estimations of $\mu _{it}$) for the setting of $\sigma _{it} / |\mu _{it}| = 1$ are shown in Fig. 4, and those for $\sigma _{it} / |\mu _{it}| = 0.5$ and 0.1 are included in the Supplementary Information (see Figs. S4, S5).

**Figure 5:**
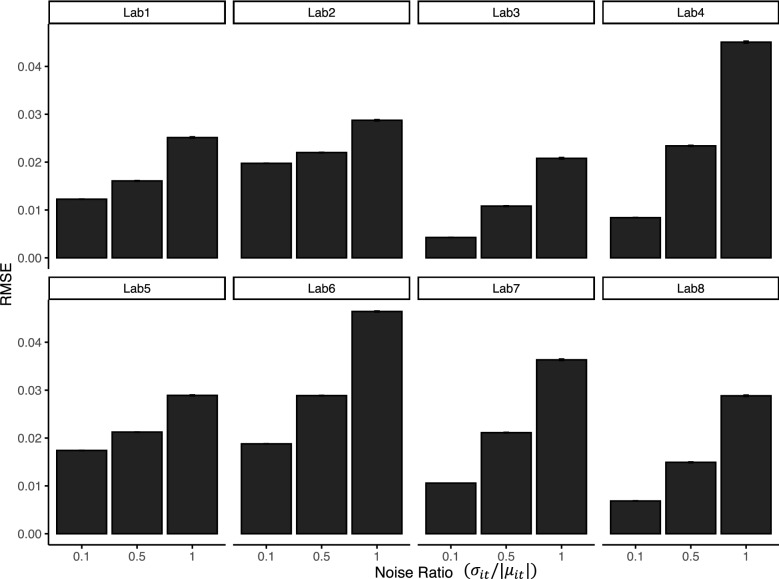
Comparing the RMSEs of the three different noise ratio settings with $\sigma _{it} / |\mu _{it}| = 0.1$, 0.5, and 1, respectively. The SEs of the RMSEs are very small compared to the RMSEs.

In Fig. 4, the red curve is the simulated true concentration values $\mu _{it}$, the blue dots are the simulated concentration values after adding noise. The truth curve is unknown to the proposed framework, and the simulated observations are used to build the black curves which successfully unveil the truth. The variation in the black curves (i.e. estimated curves) is reasonable, compared to the relatively large scale of noises. We also compare the RMSEs for three different noise ratio settings. In Fig. 5, the RMSEs are calculated by comparing the true concentrations $\mu _{it}$ with the posterior curves. When we increase the scale of noises, the RMSEs increase gradually, which suggests that the proposed framework is relatively robust to the scale of noises. The SEs of the RMSEs are very small in scale compared to the RMSEs.

## Conclusion

Unlike other studies, this study did not attempt to correlate wastewater virus concentrations with clinical cases^6,7^. Clinical surveillance has its own biases (e.g., under-reporting when prevalence is high, changing testing guidelines) and may not always be considered as an appropriate gold standard with which to compare wastewater signals. Here, we focused on exploiting exclusively the information provided by the viral concentration in wastewater along with other covariates (e.g., sample temperature, influent volume).

Relying exclusively on wastewater-based data has drawbacks because many additional factors can influence the observed virus concentrations in the WWTP samples (e.g., dilution due to rainfall or snowmelt, sample deterioration during transport, pollutants shed in wastewater affecting the RNA decay, presence of bioflim in sewer system, etc.). We currently have no access to these information in this nationwide study. However, in periods of high prevalence that overwhelm traditional clinical surveillance, wastewater-based data may be among the only data sources that can provide relatively reliable information about the state of the epidemic (as many experienced during the Omicron wave in late 2021/early 2022).

In this study, we focused on developing an framework to model the true concentration levels out of noisy and sparse observations. The proposed framework aims to answer the key question of whether an increase in the observed value indicates an actual increase in the true concentration level and if it can therefore provide accurate information on the disease burden in a community included in the catchment area of a given WWTP. For public health decision making in government agencies like PHAC, the ability to have a good grasp on dynamic COVID-19 pandemic trends is critical. The proposed framework is not only applicable to the current healthcare crisis, but it can also have broader impact on future wastewater-based epidemiology monitoring effort. As more and more resources are spent on collecting longitudinal wastewater data^24^, the proposed framework can be a perfect fit for such studies in terms of identifying and interpolating the true trajectory when researchers only have access to noisy and sparse observations.

## Supplementary Information

Supplementary Information.

## Data Availability

The data that support the findings of this study are available upon reasonable request. Correspondence should be addressed to Xiaotian Dai (xiaotian.dai@ucalgary.ca) or Thierry Chekouo (thierry.chekouotekou@ucalgary.ca).
