# Supplementary material for: Statistical framework to support the epidemiological interpretation of SARS-CoV-2 concentration in municipal wastewater
Source: Sci Rep. 2022 Aug 5;12:13490. doi: 10.1038/s41598-022-17543-y (PMC9355971; doi:10.1038/s41598-022-17543-y)
Supplement: Supplementary file 1 — Supplementary Information. [file 41598_2022_17543_MOESM1_ESM.pdf]

Supplementary Information for "Statistical Framework to Support the Epidemiological Interpretation of SARS-CoV-2 concentration in Municipal Wastewater"

Xiaotian Dai[1], David Champredon[2], Aamir Fazil[2], Chand S. Mangat[3], Shelley W. Peterson[3], Edgard Mejia[3], Xuewen Lu[1], and Thierry Chekouo[1*]

[1]Department of Mathematics and Statistics, University of Calgary, Calgary, Alberta, Canada

[2]Public Health Risk Sciences Division, National Microbiology Laboratory, Public Health Agency of Canada, Guelph, ON, Canada

[3]One Health Division, National Microbiology Laboratory, Public Health Agency of Canada, Winnipeg, MB, Canada

[*]Corresponding author: thierry.chekouotekou@ucalgary.ca

1 Additional figures

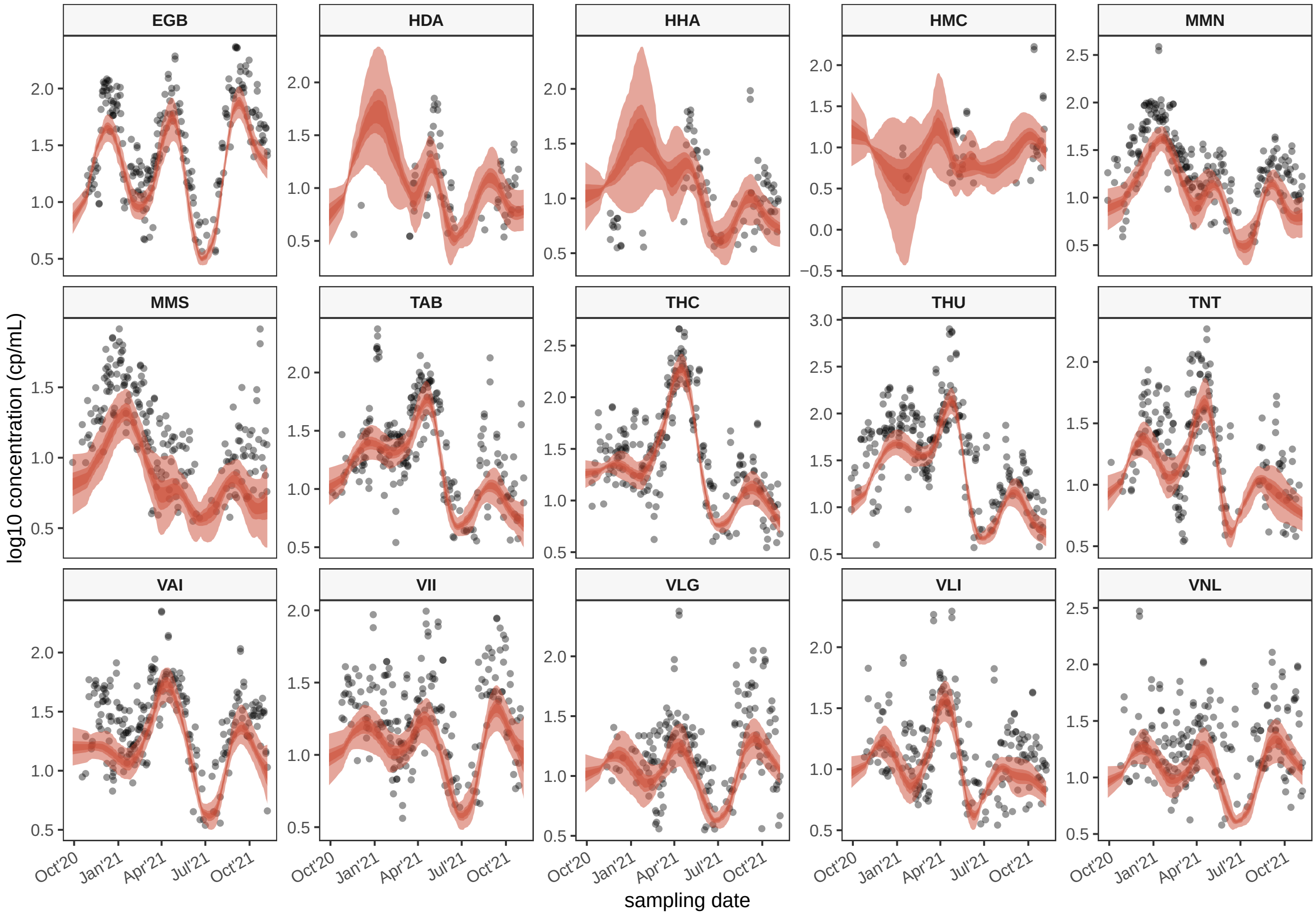

Figure S1. Black open circles represent the Log10 transformation of SARS-CoV-2 concentration observations for gene N2. Red shaded areas represent the range (lightest area), 50% (darkest area) and 80% credible intervals of posterior curves at each date, from the model with covariates. Each panel represents a sampling location.

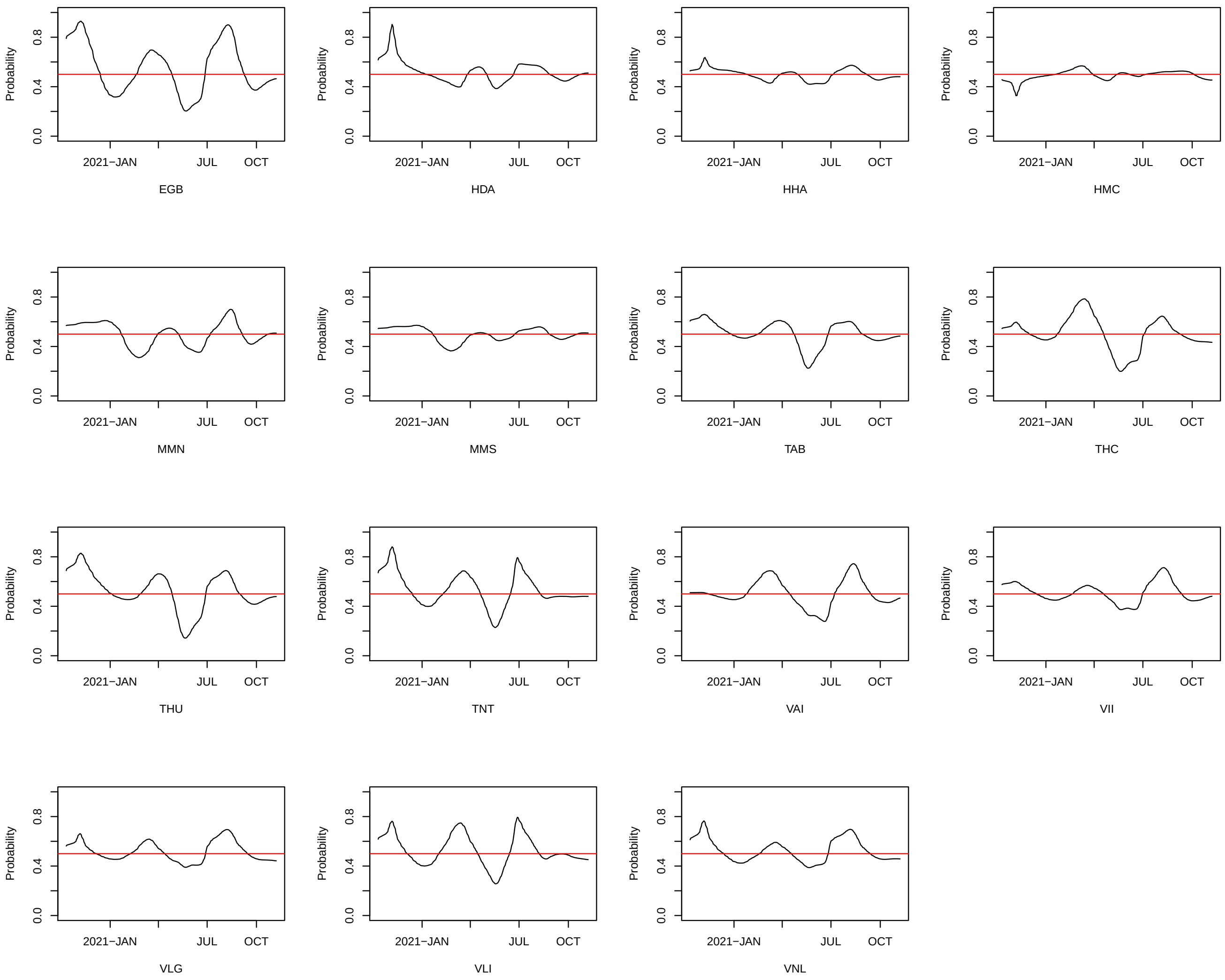

Figure S2. $Proba(\hat{Y}_i(T_{it}) > \hat{Y}_i(T_{i,t-1}))$ for the model with covariates.

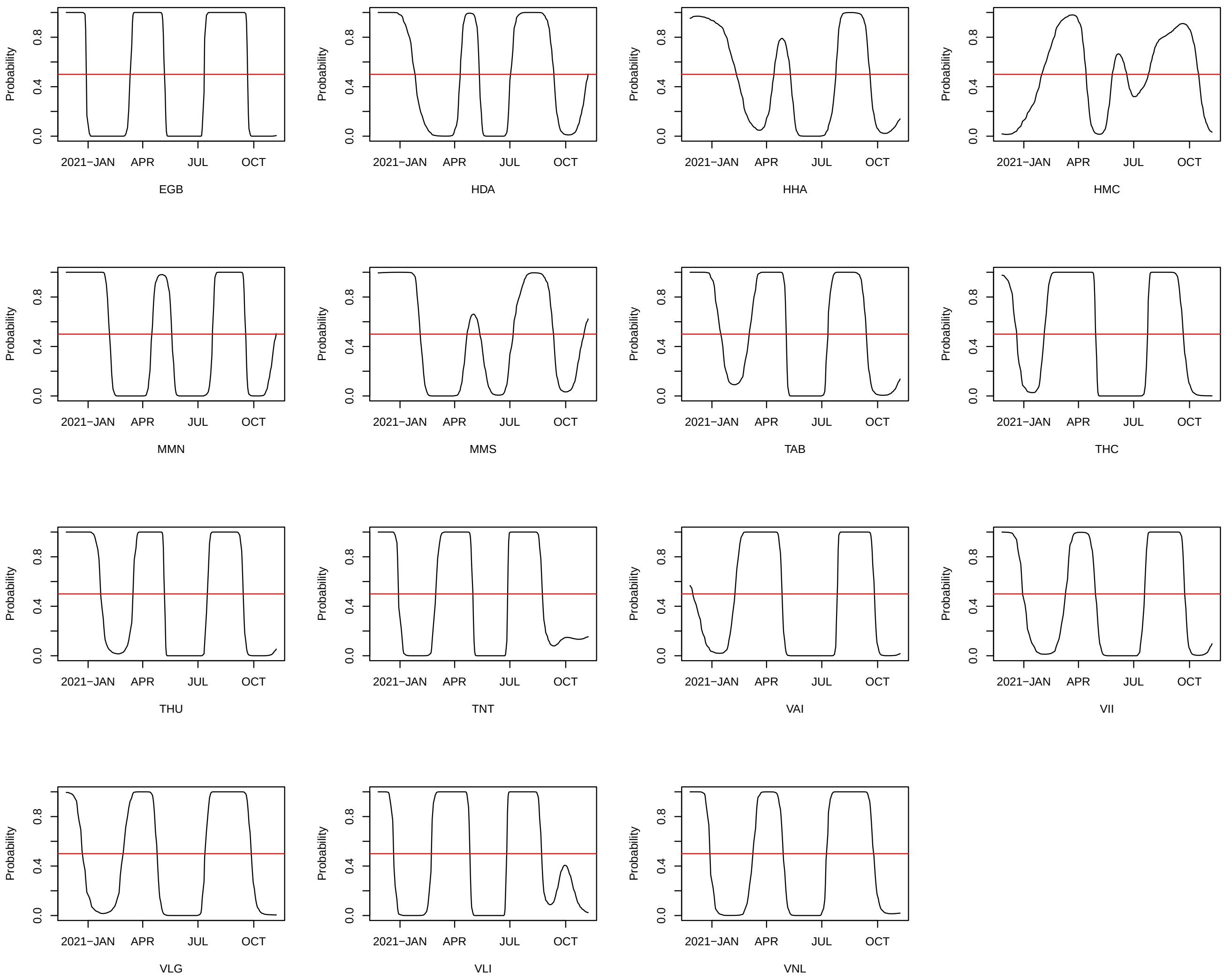

Figure S3. $Proba(\hat{Y}_i(T_{it}) > \hat{Y}_i(T_{i,t-20}))$ for the model with covariates. The time difference is about three weeks.

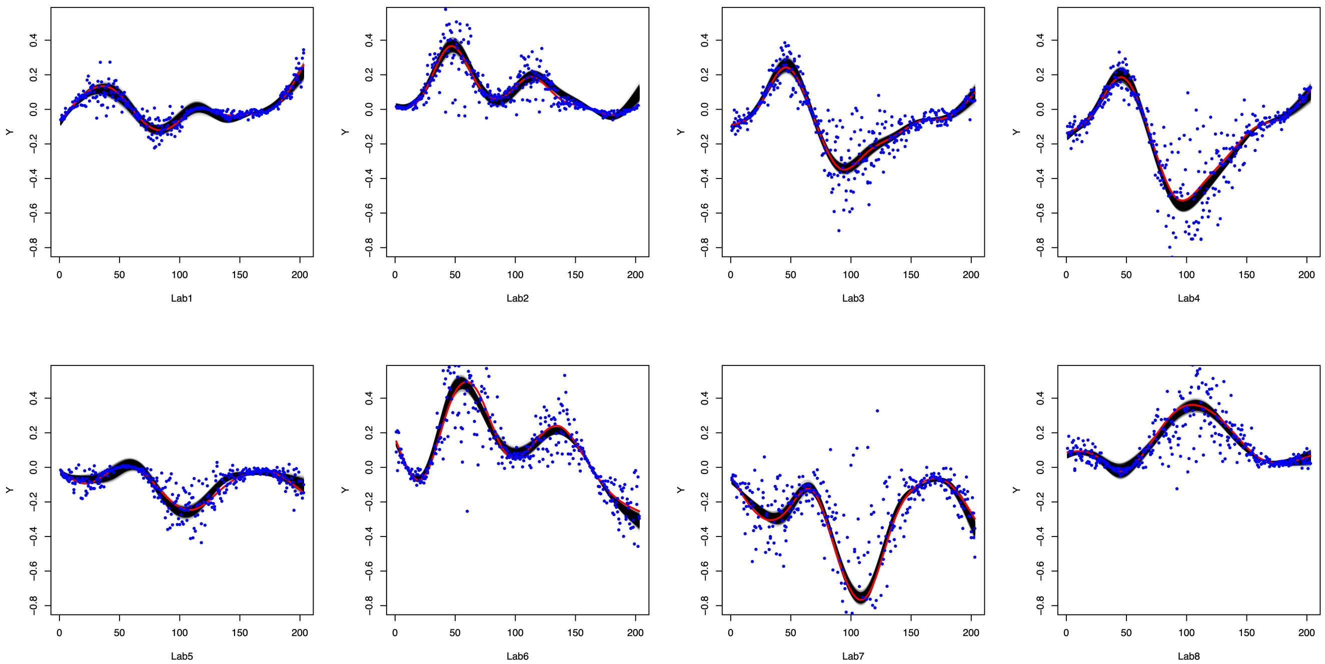

Figure S4. The simulation results of the statistical design in the main manuscript with $\sigma_{it}/|\mu_{it}| = 0.5$. The posterior curves are in black, the truth line is a red line, and the truth plus noises (simulated observations) are blue dots.

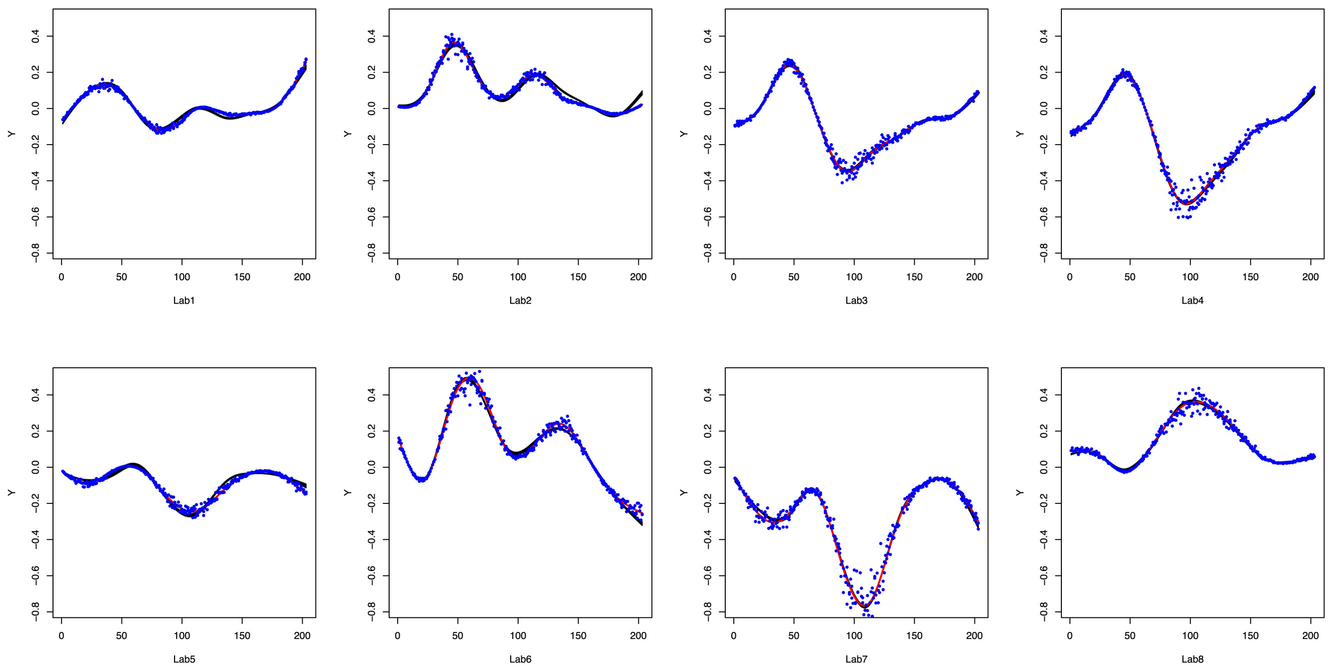

Figure S5. The simulation results of the statistical design in the main manuscript with $\sigma_{it}/|\mu_{it}| = 0.1$. The posterior curves are in black, the truth line is a red line, and the truth plus noises (simulated observations) are blue dots.

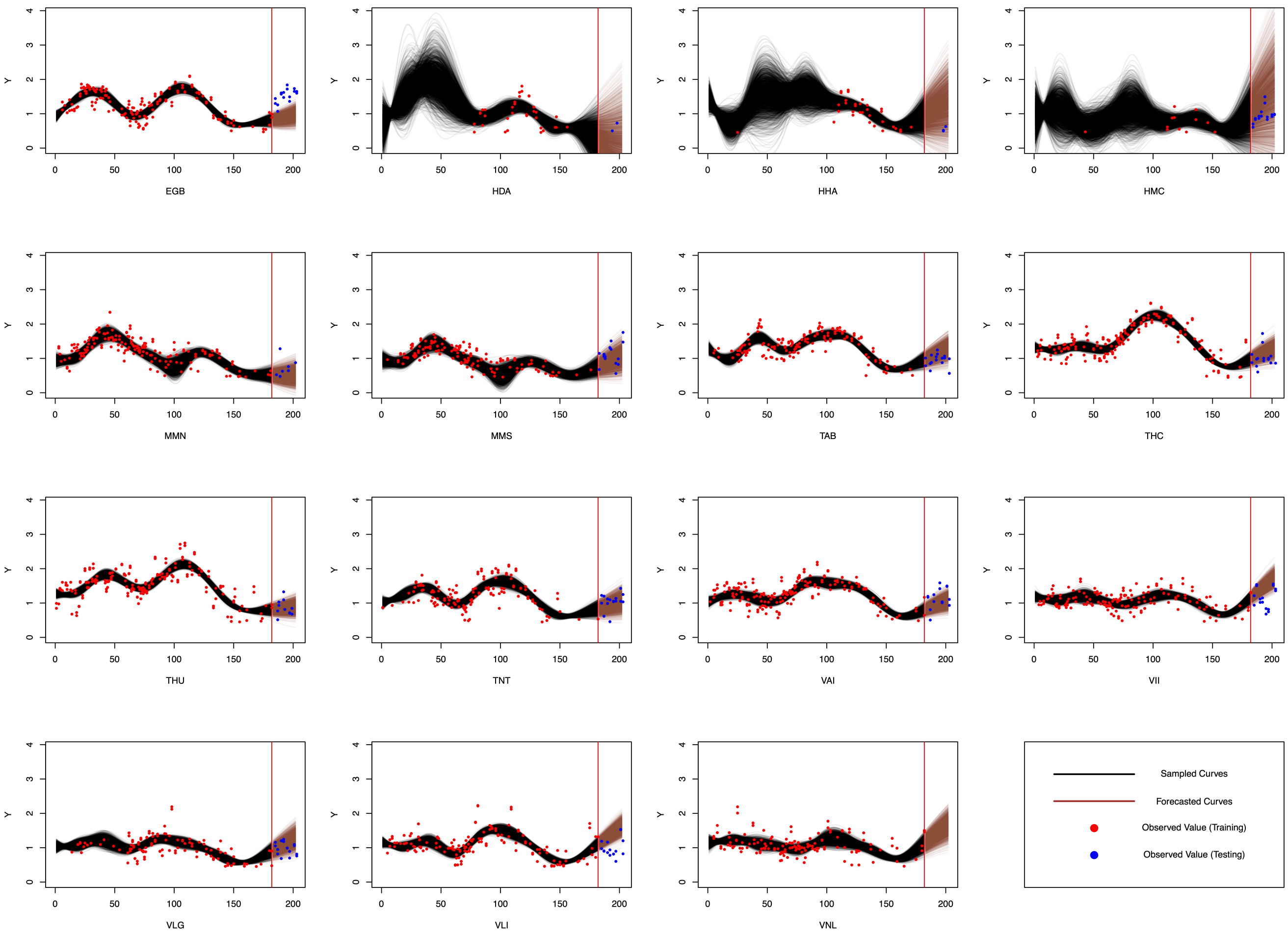

Figure S6. SARS-CoV-2 concentrations of gene N1 in wastewater in August 1st, 2021 are used as testing data and forecasted. The historic data observed before August 1st, 2021 are used as training data and used to build the full model. The posterior curves are in black, the forecasted curves are in brown, the observed values in the training period are in red, and the observed values in the testing period are in blue. The forecasting horizon is one calendar month.

2 Epidemic simulation design

This design uses a epidemic model to simulate pathogen concentration in municipal wastewater, as documented in Nourbakhsh et al. (1). The simulated data contain eight sites ($I = 8$) and virus concentration values from 200 time points ($T = 200$). At each site and each time point, two replicates of measurements are made. Also, each simulated observed concentration has an 5% probability to be missing. See Figure S7 for the simulated observations and the modeling results.

According to Nourbakhsh et al. (1), the true viral concentration entering the wastewater is simulated using the epidemic model's compartments and parameters for the SARS-CoV-2 RNA transmission and disease outcome (see Table 1 of Nourbakhsh et al. (1) for detailed descriptions). The observed SARS-CoV-2 RNA concentration is noisy and lagged behind the true concentration due to many reasons: sample transportation, laboratory processing time and reporting lags, different experimental techniques to name a few. The epidemic model is designed to mimic the real-life situations.

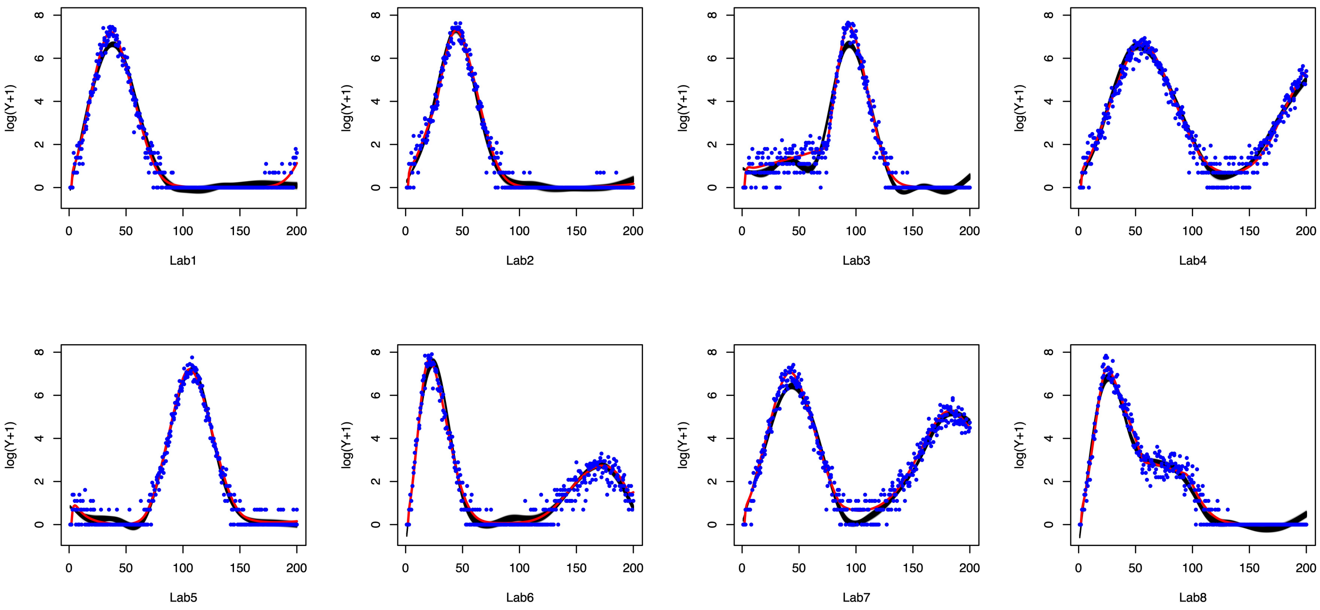

Figure S7. The simulation results of the epidemic model design. The posterior curves are in black, the truth line is a red line, and the truth plus noises (simulated observations) are blue dots.

References

1. Nourbakhsh, S. *et al.* A wastewater-based epidemic model for SARS-CoV-2 with application to three Canadian cities. *medRxiv* (2021).
